# Effect of Amino Acid Supplementation on Iron Regulation after Endurance Exercise

**DOI:** 10.3390/nu15234924

**Published:** 2023-11-25

**Authors:** Chao-An Lin, Nanako Hayashi, Claire E. Badenhorst, Kazushige Goto

**Affiliations:** 1 Graduate School of Sport and Health Science, Ritsumeikan University, Shiga 525-8577, Japan; gr0520ep@ed.ritsumei.ac.jp; 2 Department of Exercise Physiology, Nippon Sport Science University, Tokyo 158-8508, Japan; sh0108hx@outlook.com; 3 School of Sport, Exercise and Nutrition, College of Health, Massey University, Private Bag 102904, Auckland 0745, New Zealand; c.badenhorst@massey.ac.nz

**Keywords:** iron regulation, hepcidin, interleukin-6, amino acid supplementation

## Abstract

The purpose of this study was to determine the effects of pre-exercise amino acid (AA) supplementation on post-exercise iron regulation. Ten healthy males participated under two different sets of conditions in a randomized, double-blind, crossover design with a washout period of at least 21 days. Participants received either an AA supplement or placebo (PLA) for five consecutive days (4 g/dose, 3 doses/day). On the sixth day, participants ran on a treadmill for 60 min at 70% of maximal oxygen consumption ($\overset{˙}{V}$O_2max_). Venous blood samples were collected before (baseline), immediately after, and 1 and 3 h after exercise. The serum hepcidin levels increased significantly 3 h post-exercise in both trials when compared to the baseline (*p* < 0.001), but the levels were not different between trials. The plasma interleukin-6 (IL-6) level significantly increased immediately after exercise compared to the baseline (*p* < 0.001) and was significantly higher in the AA trial than in the PLA trial (*p* = 0.014). Moreover, the exercise-induced increase in serum glycerol level was significantly higher in the AA trial (21.20 ± 3.98 mg/L) than in the PLA trial (17.28 ± 4.47 mg/L, *p* = 0.017). No significant differences were observed between the AA and PLA trials for serum iron, ferritin, and total ketone body levels (*p* > 0.05). In conclusion, five days of AA supplementation augmented exercise-induced increases in IL-6 and glycerol in healthy males. However, it did not affect post-exercise iron status or regulation.

## 1. Introduction

Iron plays an important role in the transport of oxygen to working muscles during exercise, and adequate iron levels are essential for optimal physical performance [1]. Despite iron deficiency being associated with decreases in endurance capacity [2,3], the diagnosis of iron deficiency is relatively common among endurance athletes [4,5]. High incidence of iron deficiency is not limited to female athletes, and male athletes also present a higher rate compared to the general population (non-athletes).

The high prevalence of iron deficiency in athletes is likely due to exacerbated exercise-related iron loss, which cumulatively results from intestinal bleeding, hemolysis, and sweating [6]. Attention to hepcidin as a factor contributing to iron deficiency in endurance athletes has been growing for the last decade [7]. Hepcidin is a hepatocyte-derived hormone and a key regulator of iron homeostasis [8]. The primary action of hepcidin is to bind to ferroportin located on the cell surface of macrophages and enterocytes in the duodenum and hepatocytes. Ferroportin is internalized and degraded after binding, which leads to reductions in iron absorption by enterocytes and iron recycling by macrophages [9,10].

Several studies have shown that strenuous exercise increases serum hepcidin levels post-exercise [11,12]. Moreover, the increase in post-exercise hepcidin has been reported to be driven by the inflammatory cytokine interleukin-6 (IL-6) [13]. The exercise-induced increase in IL-6 appears to be further exacerbated when the muscle glycogen levels at the onset of exercise are low or have not been replenished following previous exercise [14]. Conversely, sufficient energy intake and high muscle glycogen levels before exercise have been shown to attenuate the exercise-induced increase in IL-6 [15]. Carbohydrate ingestion has been shown to reduce post-exercise IL-6. However, effects on plasma hepcidin or iron concentration were not observed [16].

Many researchers have focused on the effects of amino acid (AA) supplementation on muscle protein synthesis, exercise tolerance, and performance [17]. Branched-chain amino acids (BCAA), consisting of leucine, isoleucine, and valine, have a role in energy production by modulating the exercise-induced oxidation of serum BCAA [18]. Additionally, BCAA supplementation reduces the level of indirect muscle damage markers and muscle soreness following exercise [19]. Leucine-enriched essential AAs have been shown to alleviate IL-6 expression and impede the infiltration of inflammatory cytokines following eccentric contractions in an animal model [20]. In human studies, leucine-enriched essential AA supplementation before and after intense exercise (four × 12 min basketball game quarters and 20 min interval training) reduced IL-6 levels and muscle soreness in wheelchair basketball players [21]. Additionally, oral cystine and glutamine supplementations were reported to attenuate exercise-induced intestinal barrier dysfunction that might be associated with systemic inflammatory effects [22]. However, little has been reported on the effect of AA supplementation on IL-6 levels and subsequent iron regulation after endurance exercise.

Therefore, the purpose of this study was to determine the effects of AA supplementation on the inflammatory response and iron regulation after a single bout of endurance exercise. We hypothesized that AA supplementation would inhibit IL-6 production and attenuate the increases in exercise-induced hepcidin levels.

## 2. Materials and Methods

### 2.1. Participants

Ten healthy male participants (mean ± standard deviation [SD], age: 23 ± 2 years, height: 173.4 ± 3.8 cm, body weight: 66.0 ± 9.7 kg) were included in the present study. None of the participants were classified as anemic or iron-deficient before or during the experiment. The inclusion criteria were $\overset{˙}{V}$O_2max_ > 40 mL/kg/min; normal iron status (serum ferritin > 20 μg/L); could finish >10 km or 60 min of running; not taking medications or iron supplements; and non-smoker. All participants were informed of the purpose of the study, the experimental procedures, and the possible risks involved, and written informed consent was obtained. This study was approved by the Ethical Committee for Human Experiments at Ritsumeikan University, Japan (BKC-LSMH-2021-045), and was conducted following the Declaration of Helsinki.

### 2.2. Experimental Design

This study used a randomized, double-blind, cross-over design. All participants received the AA or placebo (PLA) treatment for 5 days (4 g/dose, 3 doses/day) [21,22]. Both trials commenced with a $\overset{˙}{V}$O_2max_ (for determination of running speed on day 6), followed by a 5-day supplementation period (days 1–5), and concluded with a single bout of endurance exercise on day 6. There were at least 21 days (32 ± 6 days) between trials as a washout period.

**Figure 1:**
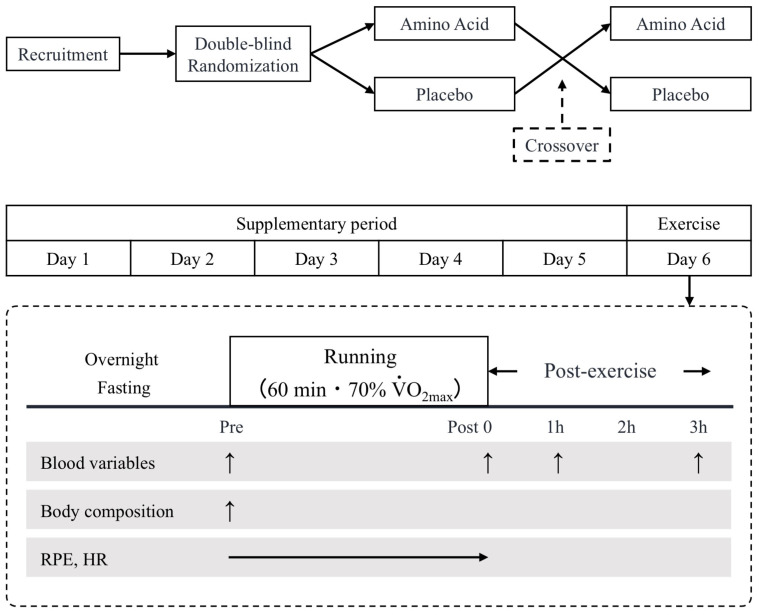
Experimental design.

Prior to each exercise trial, participants were asked to abstain from strenuous exercise for 24 h. On the main trial day (day 6), they arrived at the laboratory following an overnight fast. Exercise testing on day 6 in both trials (AA and PLA) started at 08:00–09:00 to minimize the effects of diurnal variations on IL-6 and hepcidin levels [23,24]. After a 10 min rest, a 10 mL venous blood sample was collected from an antecubital vein, and body composition was measured using a multifrequency impedance technique (InBody770; InBody Japan Inc., Tokyo, Japan). The participants performed 60 min of treadmill running at 70% of $\overset{˙}{V}$O_2max_. During each trial, the participants provided a rating of perceived exertion (RPE) every 15 min and heart rate was monitored continuously. After completing the exercise, the participants remained in the laboratory for 3 h. Three additional blood samples were collected immediately, as well as 1 and 3 h after exercise. Water intake was allowed ad libitum throughout all sessions, with matched intake between trials (Figure 1).

### 2.3. Amino Acid Supplementation

Table 1 presents the component of supplementations. The AA supplement (Ajinomoto Co., Inc., Tokyo, Japan) contained 3.8 g of a mixture of nine essential AAs (leucine, 1.028 g; isoleucine, 0.274 g; valine, 0.283 g; threonine, 0.238 g; histidine, 0.044 g; lysine, 0.429 g; methionine, 0.084 g; tryptophan, 0.018 g; and phenylalanine, 0.173 g), and two non-essential AAs (glutamine, 1.0 g; cysteine, 0.230 g) per pack. The PLA contained 4.0 g of maltodextrin per pack.

### 2.4. Experimental Protocol

#### 2.4.1. Maximal Oxygen Uptake ($\overset{˙}{V}$O_2max_)

An incremental running test was conducted on a treadmill following a 3 min warm-up at 8 km/h. The initial speed was 10 km/h, which was progressively increased by 2.0 km/h every 2 min until 14 km/h was reached. Once speed reached 14 km/h, it was increased by 0.6 km/h every 1 min until voluntary exhaustion [25]. Respiratory gases were measured during the test using an automatic gas analyzer (AE300S; Minato Medical Science, Tokyo, Japan). The data were averaged every 30 s.

#### 2.4.2. Supplement and Dietary Prescriptions

During days 1–5, participants consumed the AA or PLA supplements three times daily (every morning, at 3 pm, and at bedtime) [21]. The packaging of both supplements was identical. To mask the bitter flavor of the AAs, the supplements in both trials were ingested with 160 mL of orange juice. On days 3–5, meals were standardized and provided as commercially pre-packaged food (Nissin Healthcare Food Service Co., Ltd., Tokyo, Japan). Participants were instructed to eat the meals at their preferred times throughout the day. During the supplementation period, participants consumed only the prescribed diet and were instructed to avoid strenuous exercise. Water intake was allowed ad libitum. All meals were individually planned by a registered dietitian to confirm that all nutrients matched the Dietary Reference Intakes for Japanese 2020. The physical activity level was classified as low to medium, and energy intake was calculated with the following formula: energy intake (kcal/day) = body mass (kg) × 38.5 (kcal/kg BW/day). The macronutrient ratio was 15% protein (1.3 ± 0.1 g/kg BW/day), 25% fat, and 60% carbohydrates (energy and carbohydrate intake from supplement and co-ingested orange juice were included).

#### 2.4.3. Blood Sample Collection and Analyses

Following an overnight fast, the participants visited the laboratory at 08:00 and rested for 10 min before the first blood sample collection. Blood samples were collected before, and 0, 1, and 3 h after exercise sessions using serum separation tubes (for serum samples), tubes containing EDTA-2Na, and tubes containing EDTA-2K (for plasma and whole blood samples). Serum and plasma samples were centrifuged (10 min, 3000 rpm, 4 °C) and stored at −80 °C until subsequent analysis. Whole blood samples were stored in the refrigerator and sent to a clinical laboratory (SRL Inc., Tokyo, Japan).

Blood glucose and lactate levels were measured immediately after blood collection using a blood glucose analyzer (Freestyle; Nipro Co., Osaka, Japan), and a lactate analyzer (Lactate Pro; Arkray Co., Kyoto, Japan). Serum ferritin, iron, and total ketone body (acetoacetic acid and β-hydroxybutyric acid) were measured in the serum samples, and a complete blood count (white blood cells, red blood cells, hemoglobin, hematocrit, mean corpuscular volume (MCV), mean corpuscular hemoglobin (MCH), mean corpuscular hemoglobin concentration (MCHC), and platelet) from whole blood samples were measured in a clinical laboratory (SRL Inc.). Serum hepcidin and glycerol, as well as plasma IL-6 levels were analyzed using enzyme-linked immunosorbent assay (ELISA) kits (R&D Systems, Minneapolis, MN, USA; Cayman Chemical, Ann Arbor, MI, USA). The intra-assay coefficients of variation were 3.87%, 4.92%, and 3.56% (hepcidin); 0.88% and 1.19% (glycerol); and 2.00% and 2.88% (IL-6).

### 2.5. Statistical Analysis

All experimental data are shown as mean and standard deviation (±SD). Time-course changes in blood variables were evaluated using two-way analysis of variance (ANOVA) with repeated measures. Interaction effect (trial × time) and main effects were analyzed. When a significant interaction or main effect was detected, a post hoc Tukey–Kramer test was performed to determine where specific trial differences existed. Statistical significance was accepted when *p*-value < 0.05. Partial eta squared (*η*_p_^2^) was calculated as a measure of effect size for data analyzed by two-way ANOVA with repeated measures. Cohen’s *d* was calculated for paired *t*-tests.

## 3. Results

### 3.1. Body Composition and Running Speed during Exercise

The mean height of the participants was 173.4 ± 3.8 cm. The mean body mass was 65.8 ± 9.1 kg for the AA trial and 65.5 ± 8.4 kg for the PLA trial, and the respective body fat percentages were 15.2 ± 3.8% and 14.9 ± 3.3%. These values did not differ between trials (*p* > 0.05). The running speeds were 10.1 ± 0.8 km/h (AA trial) and 10.1 ± 0.9 km/h (PLA trial) (*p* > 0.05).

### 3.2. IL-6 and Hepcidin

**Figure 2:**
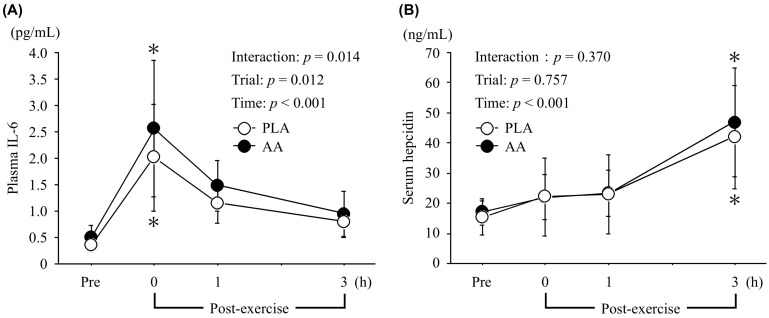
Changes in plasma IL-6 (**A**) and serum hepcidin (**B**) levels before and after exercise. Values are means ± SD. *; *p* < 0.05 vs. Pre.

Exercise-induced changes in plasma IL-6 and serum hepcidin are shown in Figure 2. The plasma IL-6 level increased immediately after exercise in both trials (main effect of time, *η*_p_^2^ = 0.739, *p* < 0.001). There was an interaction effect (*η*_p_^2^ = 0.322, *p* = 0.014) and a main effect of trial (*η*_p_^2^ = 0.519, *p* = 0.012). Moreover, the area under the curve (AUC) for the plasma IL-6 levels during and post-exercise was significantly higher in the AA trial (5.98 ± 2.29 pg/mL∙240 min) than the PLA trial (4.69 ± 1.70 pg/mL∙240 min, *p* = 0.011, *d* = 0.64).

There was a main effect of time for the serum hepcidin level (*η*_p_^2^ = 0.808, *p* < 0.001), but there was no interaction effect (*η*_p_^2^ = 0.108, *p* = 0.370) or main effect of trial (*η*_p_^2^ = 0.027, *p* = 0.757).

### 3.3. Serum Ferritin, Iron, Blood Glucose, and Lactate Levels

Table 2 presents the changes in serum ferritin, iron, blood glucose, and lactate levels before and after exercise. Baseline serum ferritin levels did not differ between the AA (101.1 ± 59.5 ng/mL) and PLA (92.2 ± 56.9 ng/mL) trials (*d* = 0.15, *p* = 0.089). A main effect of time (*η*_p_^2^ = 0.434, *p* = 0.003) was detected, but there was no interaction effect (*η*_p_^2^ = 0.043, *p* = 0.753) and no main effect of trial (*η*_p_^2^ = 0.308, *p* = 0.076).

For the serum iron level, there was no interaction effect (*η*_p_^2^ = 0.198, *p* = 0.163) or main effect of trial (*η*_p_^2^ = 0.016, *p* = 0.710). A main effect of time was observed (*η*_p_^2^ = 0.536, *p* = 0.001), but there was no significant difference among trials at any time point.

For blood glucose levels, there was no interaction effect (*η*_p_^2^ = 0.073, *p* = 0.558), no main effect of time (*η*_p_^2^ = 0.114, *p* = 0.343), and no main effect of trial (*η*_p_^2^ = 0.080, *p* = 0.400). For blood lactate levels, there was no interaction effect (*η*_p_^2^ = 0.019, *p* = 0.912), no main effect of time (*η*_p_^2^ = 0.108, *p* = 0.337), and no main effect of trial (*η*_p_^2^ = 0.001, *p* = 0.926).

### 3.4. Serum Glycerol and Total Ketone Body Levels

**Figure 3:**
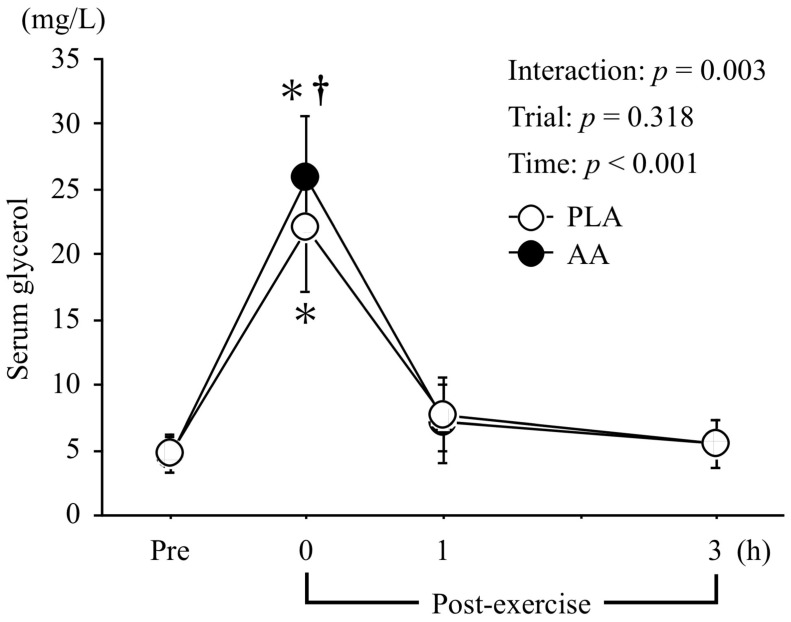
Changes in serum glycerol levels. Values are means ± SD. *; *p* < 0.05 vs. Pre. †; *p* < 0.05 vs. PLA.

Changes in the serum glycerol levels are shown in Figure 3. There was an interaction effect (*η*_p_^2^ = 0.396, *p* = 0.003) and a main effect of time (*η*_p_^2^ = 0.960, *p* < 0.001), but no main effect of trial (*η*_p_^2^ = 0.111, *p* = 0.318). Serum glycerol levels were significantly higher immediately after exercise in the AA than the PLA trial (*p* < 0.05).

**Figure 4:**
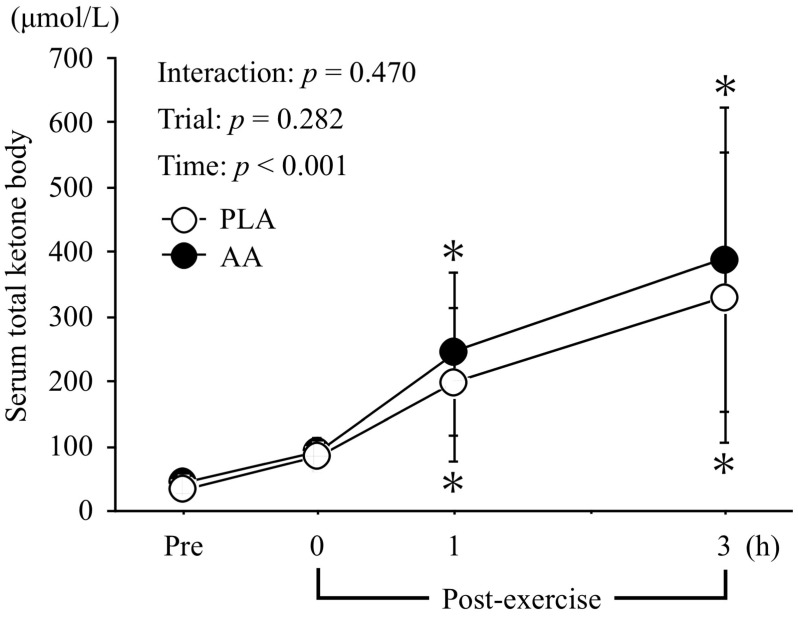
Changes in serum total ketone body levels before and after exercise. Values are means ± SD. *; *p* < 0.05 vs. Pre.

Figure 4 presents the changes in serum total ketone body levels. There was a main effect of time for total ketone body levels (*η*_p_^2^ = 0.691, *p* < 0.001), but no interaction effect (*η*_p_^2^ = 0.073, *p* = 0.470) or main effect of trial (*η*_p_^2^ = 0.147, *p* = 0.282).

## 4. Discussion

In the present study, we determined the effect of five days of AA supplementation on iron regulation after a single session of endurance exercise. The main finding was that five days of AA supplementation did not attenuate the exercise-induced increase in hepcidin. However, AA supplementation was associated with augmented IL-6 and glycerol levels post-exercise.

Serum hepcidin levels increased significantly 3 h after endurance exercise in both trials. This result is consistent with previous studies that have reported transient increases in the serum hepcidin level 3–6 h after acute endurance exercise [12,13]. However, no differences were found in the post-exercise response between trials. In the present study, we provided 3 days of standardized (identical) diet with sufficient energy intake to meet the Japanese RDA during both trials. The standardization of the diet is likely to have limited any impact that energy and carbohydrate availability may have on hepcidin levels post-exercise, factors that have been previously associated with exacerbated hepcidin levels post-exercise [26,27]. It should be noted that the magnitude of the post-exercise increase in hepcidin has been shown to be primarily determined by basal iron stores [7]. In the present study, the 3 h post-exercise serum hepcidin level was significantly higher in participants with a baseline serum ferritin level >100 ng/mL (*n* = 7) compared with those with levels of 30–100 ng/mL (*n* = 13, *p* = 0.04). However, no significant difference in baseline serum ferritin level was detected between trials. Therefore, with no difference in baseline and post-exercise serum ferritin or iron levels between both trials, similar elevations in hepcidin post-exercise are somewhat expected.

Exercise-induced IL-6 has been associated with increases in serum hepcidin levels post-exercise [9,28]. We hypothesized that 5 days of AA supplementation would attenuate the exercise-induced increase in IL-6. However, the AA trial resulted in a significantly higher IL-6 level than the PLA trial. To ensure we matched running intensities between trials in the present study, we determined $\overset{˙}{V}$O_2max_ at the start of each trial. This was performed because previous research has reported that running intensity correlates significantly with an increase in plasma IL-6 [29]. Since no significant differences in heart rate (*p* = 0.764) or relative perceived exertion (*p* = 0.543) were detected during the 60 min exercise, we are confident that running intensity and physiological strain did not differ between the trials and would not be a contributing factor to differences in IL-6 levels between the trials.

In the present study, IL-6 was the only inflammatory marker [30,31] that was measured. However, in the absence of other inflammatory cytokines (e.g., IL-1β and tumor necrosis factor-α), the authors have noted that serum ferritin, an acute phase reactant [32,33], was not different between trials. Therefore, differences in exercise-induced inflammation may not be the primary factors contributing to differences in IL-6 levels between trials in this study.

As well as having an inflammatory effect, IL-6 is a myokine secreted from working muscles in response to an energy deficit and plays a role in promoting catabolism through lipolysis [34]. van Hall et al. (2003) reported that IL-6 stimulated lipolysis and fat oxidation in humans [35], with subsequent research demonstrating IL-6 as regulator of lipid metabolism during a marathon [36]. In addition, Ma et al. (2022) reported that 7-day supplementation with a non-essential AA (cystine/glutamine) mixture enhanced fatty acid utilization; however, changes in IL-6 were not reported [37]. In the present study, exercise-induced increases in serum glycerol in the AA trial were observed, suggesting an increase in lipolysis. This increase in glycerol in the AA trial may be attributable to the higher IL-6 level. Therefore, the augmented increase in IL-6 observed during the AA trial may be reflective of increased metabolic requirements, but not associated with an inflammatory response. However, future research may need to consider investigating the causal mechanism of IL-6 increases with AA supplementation and the subsequent association with lipolysis.

There were potentially confounding factors associated with the outcomes of the present study. Sleep deprivation augmented markers of inflammation [38]. However, previous research demonstrated that a single night of partial sleep deprivation did not affect exercise-induced IL-6 and hepcidin levels [39]. Individual differences in inflammatory and iron regulation parameters were also considered. However, we utilized a crossover design to minimize inter-individual variations. Seasonal factors and changes in lifestyle rhythm may also have had an affect, but two trials were completed within two months with at least a 21-day washout period (32 ± 6 days).

The present study had some limitations that should be discussed. First, we used maltodextrin as a PLA supplement. Several studies have shown that carbohydrate intake affects IL-6 production [15,27,40]. However, because the maltodextrin we utilized was a low-dose treatment (12 g/day, 60 g in total for 5 days), and the participants did not ingest either AA supplementation nor PLA on the day of exercise, the effect of the PLA on exercise-induced increases in IL-6 and hepcidin would be minor. Second, we used a single 60 min running test with an exercise intensity of 70% of $\overset{˙}{V}$O_2max_. However, for the given intensity, previous research has suggested that prolonged exercise results in greater hepcidin levels (60% of $\overset{˙}{V}$O_2max_, 120 min) [11]. Therefore, the impact of AA supplementation on iron metabolism when exercising for a longer duration and/or at a higher intensity or on consecutive days (e.g., during a training camp) has not been fully elucidated. Long-term effects of amino acid supplementation may need further analyses in future studies. In addition, all measurements obtained during and after exercise in this study were conducted under fasting conditions, so further analyses under fed conditions may be necessary. Finally, the present study recruited male participants for the purposes of examining nutritional strategies that could enhance iron status without the use of iron supplements. However, iron deficiency has been reported to be more common in female athletes (~15–35%) compared with male athletes (5–11%) [41]. Therefore, the effect of AA supplementation on iron regulation in female participants would be a valuable topic for future study.

## 5. Conclusions

The present study demonstrated that 5 days of AA supplementation augmented exercise-induced increases in IL-6 and glycerol levels in healthy males. Moreover, 60 min of running significantly increased the serum hepcidin level, but the exercise-induced elevation was not affected by AA supplementation. Furthermore, no significant differences in serum iron or ferritin levels were detected following the endurance exercise. These findings suggest that 5 days of AA supplementation do not affect post-exercise iron regulation following a single bout of endurance exercise.

## Figures and Tables

**Table 1:** Component of supplementation.

Ingredient	Amino Acid (AA)		Placebo (PLA)	
	Compound Ratio (%)	Amount Per Serving (g)	Compound Ratio (%)	Amount Per Serving (g)
L-Leucine	23.37	1.03	0.00	0.00
L-Isoleucine	6.22	0.27	0.00	0.00
L-Valine	6.43	0.28	0.00	0.00
L-Glutamine	22.73	1.00	0.00	0.00
L-Cystine	5.23	0.23	0.00	0.00
Other essential amino acid	22.41	0.99	0.00	0.00
Other ingredients (e.g., carbohydrate, water)	13.61	0.60	100.00	4.00

**Table 2:** Serum ferritin, iron, blood glucose, and lactate levels before and after exercise.

	Trial	Pre	Post-Exercise		
			Post 0	Post 1	Post 3 (h)
Ferritin (ng/mL)	AA	101.1 ± 59.5	107.1 ± 61.1	105.7 ± 60.2	105.9 ± 60.5
	PLA	92.2 ± 56.9	96.9 ± 59.4	95.7 ± 60.5	97.1 ± 57.8
Iron (μg/dL)	AA	136 ± 30	157 ± 30	159 ± 28	152 ± 25
	PLA	139 ± 33	158 ± 36	150 ± 30	148 ± 30
Glucose (mg/dL)	AA	84 ± 4	86 ± 6	82 ± 6	81 ± 3
	PLA	84 ± 8	85 ± 10	84 ± 8	84 ± 6
Lactate (mmol/L)	AA	1.9 ± 0.4	2.1 ± 1.1	1.7 ± 0.4	1.7 ± 0.4
	PLA	1.9 ± 0.5	2.1 ± 0.9	1.8 ± 0.3	1.7 ± 0.2

Values are means ± SD.

## Data Availability

The data presented in this study are available on request from the corresponding author. The data are not publicly available due to ethical restrictions.
